# Alleviation of Cerebral Infarction of Rats With Middle Cerebral Artery Occlusion by Inhibition of Aquaporin 4 in the Supraoptic Nucleus

**DOI:** 10.1177/1759091420960550

**Published:** 2020-09-26

**Authors:** Dan Cui, Shuwei Jia, Jiawei Yu, Dongyang Li, Tong Li, Yang Liu, Jinlong Chang, Xiaoran Wang, Xiaoyu Liu, Yu-Feng Wang

**Affiliations:** 1 Department of Physiology, School of Basic Medical Sciences, Harbin Medical University, Harbin, China; 2 The Seventh Affiliated Hospital, Sun Yat-sen Universtiy, Shenzhen, China

**Keywords:** astrocytes, glial fibrillary acidic protein, ischemic stroke, vasopressin

## Abstract

In ischemic stroke, vasopressin hypersecretion is a critical factor of cerebral swelling and brain injury. To clarify neural mechanisms underlying ischemic stroke-evoked vasopressin hypersecretion, we observed the effect of unilateral permanent middle cerebral artery occlusion (MCAO) in rats on astrocytic plasticity and vasopressin neuronal activity in the supraoptic nucleus (SON) as well as their associated cerebral injuries. MCAO for 8 hr caused cerebral infarction in the MCAO side where water contents also increased. Immunohistochemical examination revealed that the percentage of phosphorylated extracellular signal-regulated protein kinase 1/2 (pERK1/2)-positive vasopressin neurons in the SON of MCAO side was significantly higher than that in non-MCAO side and in sham group. In the cortex, pERK1/2 and aquaporin 4 expressions increased significantly in the infarction area, while glial fibrillary acidic protein (GFAP) reduced significantly compared with the noninfarction side in brain cortex. Microinjection of N-(1,3,4-Thiadiazolyl)nicotinamide-020 [TGN-020, a specific blocker of aquaporin 4] into the SON blocked MCAO-evoked increases in pERK1/2 in the SON as well as the reduction of GFAP and the increase in pERK1/2 and aquaporin 4 in the infarction area of the cortex. Finally, oxygen and glucose deprivation reduced GFAP expression and the colocalization and molecular association of GFAP with aquaporin 4 in the SON in brain slices. These effects were blocked by TGN-020 and/or phloretin, a blocker of astrocytic volume-regulated anion channels. These findings indicate that blocking aquaporin 4 in the SON may reduce the activation of vasopressin neurons and brain injuries elicited by vasopressin during ischemic stroke.

Ischemic stroke often causes cerebral brain edema that is a major pathological change in close association with vasopressin hypersecretion (Jia et al., 2016). Patients with ischemic stroke exhibit increased vasopressin levels in the blood (Barreca et al., 2001) and the cerebrospinal fluid (Widmayer et al., 2010). Blood vasopressin levels have positive correlation with the severity of brain injury (Barreca et al., 2001). By contrast, blocking vasopressin receptor can alleviate ischemic injury (Kleindienst et al., 2006; X. Liu et al., 2010; Zeynalov et al., 2015). Circulating vasopressin is mainly produced in the hypothalamo-neurohypophysial system including the supraoptic nucleus (SON). In the SON, vasopressin neurons innervate both the posterior pituitary and extrahypothalamic brain regions via axons and their collaterals, respectively. Vasopressin release depends on the excitation of vasopressin neurons (Hou et al., 2016). Thus, clarification of the regulation of vasopressin neuronal activity is an essential strategy for developing more radical therapies of ischemic stroke via suppressing vasopressin hypersecretion.

Among many factors regulating vasopressin neuronal activity, astrocytic plasticity is essential since inhibiting astrocytic metabolism with fluorocitrate (Xiong et al., 2011) or l-aminoadipic acid (Sun et al., 2016) blocked osmotic activation of vasopressin neurons in the SON. Astrocytic plasticity can largely determine the functions of other modulating factors including extracellular microenvironment, synaptic inputs, gap junctional communication, and cellular apposition through changing the presence of astrocytic processes around vasopressin neurons (Theodosis et al., 2008; Hou et al., 2016). In ischemic stroke, retraction of astrocytic processes could activate vasopressin neurons due to decreased astrocytic absorption of extracellular levels of glutamate, K^+^, and osmolytes (Jia et al., 2016). In studies using mongolian gerbils, it was found that hypothalamic vasopressin neurons were involved in the penumbra areas of the infarction (Loesch, 1983b) and that ischemia damage could evoke swelling of astrocytes in the SON (Loesch, 1983a). In ischemia-associated astrocytic plasticity, abnormal activity of glial fibrillary acidic protein (GFAP) and aquaporin 4 (AQP4) may play essential roles in abnormal neuronal activity or neural damages. GFAP provides the cytoskeleton and scaffold, and AQP4 determines cytosolic volume in the space between cell membrane and the cytoskeleton. Retraction of GFAP filaments along with decrease in AQP4 membrane localization can result in retraction of astrocytic processes, thereby promoting vasopressin neuronal activity (Wang and Parpura, 2016, 2018). However, experimental evidence supporting astrocytic involvement in the vasopressin hypersecretion and its underlying cellular mechanisms remains to be collected.

In this study, we simulated focal cerebral ischemia with unilateral middle cerebral artery occlusion (MCAO) in rats, examined astrocytic plasticity and vasopressin neuronal activity in the SON and their associated cerebral injuries. We also observed the effects of blocking AQP4 in the SON on MCAO-evoked injuries in the cerebral cortex. In addition, we observed astrocytic GFAP and AQP4 expressions in the SON of brain slices under oxygen and glucose deprivation (OGD) conditions.

## Materials and Methods

All procedures in this study were in accordance with the guidelines on the use and care of laboratory animals set by National Institutes of Health and were approved by the Institutional Animal Care and Use Committees of Harbin Medical University.

### Preparation of MCAO Model

**Figure 1.**
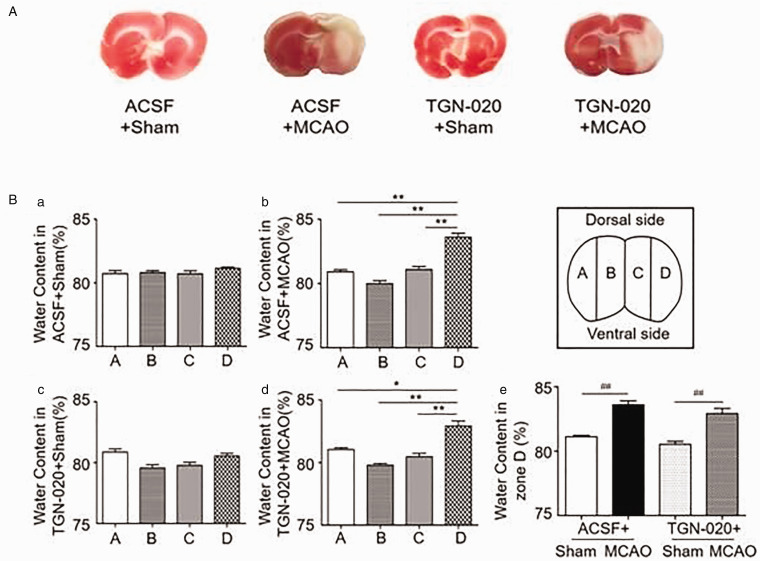
Brain Injury and Cerebral Water Content in Rats of Middle Cerebral Artery Occlusion (MCAO) and the Influence of Microinjection of N-(1,3,4-Thiadiazolyl) nicotinamide (TGN-020) in the Supraoptic Nucleus (SON). A: Representative images of 2,3,5-triphenyltetrazolium chloride (TTC) staining of the cerebral cortex in different groups: ACSF + sham, ACSF + MCAO, TGN-020 + sham, and TGN-020 + MCAO. B: Bar graphs showing cerebral water contents in different zones (inset) of the cerebral cortex of sham-operated (a, c) and MCAO rats (b, d) without (a, b) and with microinjection of TGN-020 in the SON (c, d): Zone A (*n* = 6), left temporal section (non-MCAO side); Zone B (*n* = 6), left medial section (non-MCAO side); Zone C (*n* = 6), right medial section (MCAO side), and Zone D (*n* = 6), right temporal section (MCAO side). Be: Bar graphs showing statistical analysis of cerebral water content at Zone D in different group. *p < .05, **p < .01 compared with Zone A by analysis of variance; ##*p* < .01 compared with the sham group by paired *t* test. ACSF = artificial cerebrospinal fluid.

Male Wistar rats (220–240 g) were used for this study. All rats were housed in a room of 21°C to 23°C with 12/12 hr light/dark cycle and free access to food and water. The rats were randomly divided into the sham operation and MCAO groups. The methods to prepare MCAO model were as the followings. Firstly, rats were anesthetized with 10% chloral hydrate (0.3 ml/100g) and fixed at supine position. A midline incision in the neck was made to expose the right common carotid artery and the external and internal carotid arteries, and then, the right common carotid artery and the external carotid artery were ligated. A cut on the right common carotid artery was made and a silicon-coated 4-0 nylon monofilament (diameter 0.28 mm) was inserted along the right external carotid artery to occlude the origin of the right middle cerebral artery until 8 hr later. Blood flows through the right parietal lobe irrigated by the right middle cerebral artery were monitored throughout the stages of MCAO with a blood flow meter (Omegaflo-Lab, LDF-C1, Omegawave, Inc., Tokyo, Japan). The success of MCAO was indicated by a sudden reduction of blood flow during thread insertion, which remained at that level before being sacrificed at 8 hr later (Figure 1A). Sham operation group received all the surgical procedures except the insertion of occluding thread. The whole procedures were performed in a sterile environment and the body temperature was maintained at 37 ± 0.5 °C with a heating pad. Rats were returned to their individual cages after suturing the surgical wounds. The brain and blood were sampled at 8 hr after the MCAO without reperfusion.

### Evaluation of Brain Injury and Tissue Preparation

To evaluate brain damages, we performed neurological tests at 30 min before 8 hr of MCAO. The grading of neurologic examination was a modification of the Longa’s grading system. That is, Grade 0, no observable deficit (normal); Grade 1, blepharoptosis (moderate); Grade 2, paralysis, ataxia with blepharoptosis (severe).

For assaying the area of infarction, the brain was cut into slices after dissection and then subjected to conventional staining of 2,3,5-triphenyltetrazolium chloride (TTC, Sigma-Aldrich, Shanghai, China; Figure 1B). In brief, brain slices were soaked in ice-cold artificial cerebrospinal fluid (ACSF) for 2 min. The ACSF contained (in mM) 126 NaCl, 3 KCl, 1.3 MgSO_4_, 2.4 CaCl_2_, 1.3 NaH_2_PO_4_, 26 NaHCO_3_, 10 Glucose, 305 mEq/Kg water, pH 7.4, and gassed with 95% O_2_/5% CO_2_. Then, they were cut into 2 mm-thick coronal sections sequentially in a brain module, incubated with 2% TTC at 20 °C for 20 min, fixed in 4% paraformaldehyde at room temperature for 4 hr, and then photographed for evaluating brain injuries.

To evaluate brain water contents, conventional wet/dry method was used. That is, brain tissues were trimmed to ∼200 mg blocks and then placed in a baker at 180 °C for 3 hr. The contents of brain water were quantitated by calculating the percentage of weight loss through drying.

### In Vitro OGD Study

Male adult rats were decapitated; the hypothalamus in the brain was dissected and cut into 300 µm-thick coronal brain slices in ice-cold ACSF. After adaptation in the ACSF at 32 °C for 30 min, the slices were treated with OGD, N-(1,3,4-Thiadiazolyl)nicotinamide (TGN-020, CAS 51987-99-6, a blocker of AQP4, Santa Cruz Biotechnology, Shanghai, China) or phloretin (a blocker of astrocytic volume-regulated anion channels, P7912, CAS-NO: 60-82-2, Sigma-Aldrich, Shanghai, China) in the following six groups including control, OGD, TGN-020 (10 µM), OGD + TGN-020, phloretin (30 µM), and OGD + phloretin. In OGD group, glucose was replaced with equal molar sucrose, and oxygen supplement of the ACSF was stopped at 5 min before brain slices were transferred into the reaction chamber. Slices in the control group were incubated in the ACSF at 32 °C for 45 min. Slices in OGD group were incubated in the ACSF for 15 min and then transferred into OGD-ACSF. Slices in TGN-020 or phloretin group were first incubated in the ACSF for 15 min and then transferred into ACSF containing TGN-020 or phloretin and incubated for 30 min. OGD + TGN-020 and OGD + phloretin groups were performed by transferring the slices to OGD solution with TGN-020 or phloretin and incubating for 30 min after 10 min with TGN-020 or phloretin, respectively. The OGD was applied for 30 min because bilateral common carotid artery occlusion for 10 min can cause marked swelling of perivascular astrocytes in the SON (Loesch, 1983a) and reversible swelling of astrocytes in the SON occurs within 30 min (Y. F. Wang et al., 2013). In immunohistochemistry, the treated brain slices were fixed in 4% paraformaldehyde at room temperature for 4 hr and then rinsed before further processing. For Western blots, the SON was punched out of the brain slices and then stored at −80°C for later protein extraction.

### Microinjection of TGN-020 into the SON

The methods of microinjection of agents into the SON were the same as previously reported (X. Y. Liu et al., 2017). In brief, anesthetized rats were placed in a stereotaxic apparatus (M302982, Zhimu Duobao Biotechnology Co., Ltd, Beijing, China) according to a rat brain atlas (Paxinos and Watson, 1998). After exposing the dorsal surface of the skull, 1 µl ACSF (vehicle) or ACSF containing 10 µM TGN-020 and 0.4% Trypan blue was injected into each SON bilaterally in two sites according to the atlas. The coordinates of microinjection were 0.5 to 1.5 mm posterior to the Bregma point, 1.4 to 1.8 mm lateral to the midline, and 8.6 to 8.9 mm below skull surface. Each microinjection took 10 min and then remained in situ for 5 min before the next injection. Twenty min after the injection, rats were subjected to the sham or MCAO procedures as described above. The injection sites were verified by examining the deposition of Trypan blue after the experiment.

### Western Blots

The right and left brain regions were sampled separately for identifying MCAO-associated vasopressin neuronal activity and astrocytic involvement in MCAO effects. The cerebral cortex in different zones and the SON were dissected from MCAO rats or punched out of brain slices for examining the expressions of phosphorylated extracellular signal-regulated protein kinase 1/2 (pERK1/2), GFAP, and/or AQP4. The method of Western blots was basically the same as previously reported (X. Y. Liu et al., 2017) with minor modification. In brief, tissues were homogenized to release protein in a lysis buffer (20115ES60, Yeasen, Shanghai, China) with a tissue lyser. Protein concentration was measured at 562 nm wavelength in bicinchoninic acid assay agent (20201ES76, Yeasen, Shanghai, China). After preparing SON lysate and assaying its protein concentration, 60 µg/sample of protein was loaded and separated on 10% sodium dodecyl sulfate-polyacrylamide gel electrophoresis gels. The protein was then transferred onto polyvinylidenedifluoride membranes. After blocking nonspecific binding sites with 5% bovine serum albumin in Tris-buffered saline containing 0.1% Tween 20 for 2 hr at room temperature, protein membranes were incubated with primary antibodies against target proteins at 4 °C overnight. The primary antibodies were purchased from Santa Cruz Biotechnology (sc, Shanghai, China), Omnimabs (OM, Alhambra, CA) or Abcam (ab, Cambridge, UK). They included antibodies against GFAP (sc-33673, mouse, 1:1000, RRID: AB____627673), AQP4 (ab125049, rabbit, 1:1000, RRID: AB____ 11131948), and pERK1/2 (sc-136521, mouse, 1:400, RRID: AB____10856869). Loading controls were set using antibodies against glyceraldehyde 3-phosphate dehydrogenase (OM254102, rabbit, 1:1500), ERK1/2 (sc-514302, mouse, 1:1000, RRID: AB____2571739), or β-tubulin (30302ES60, rabbit, 1:2000, Yeasen, Shanghai, China). The membranes were further processed with horseradish peroxidase-conjugated secondary antibodies (111-035-003, Peroxidase AffiniPure goat anti-rabbit immunoglobulin (Ig) G heavy and light chains (H&L), RRID: AB_2313567; 115-035-003, Peroxidase AffiniPure goat anti-mouse IgG H&L, RRID: AB_10015289, Jackson Lab, Shanghai, China) for 2 hr. Protein bands were visualized with an automated chemiluminescence imaging analysis system (Tanon 5200, Shanghai, China).

### Co-Immunoprecipitation

Methods for evaluating molecular association between GFAP and AQP4 under OGD condition were modified from a previous publication (P. Wang et al., 2019). Briefly, total tissue lysates of the SON were precleared with protein A/G agarose beads (sc-2003, RRID: AB_10201400) to reduce nonspecific binding. To form immunocomplex, immunoprecipitating antibody against GFAP (sc-33673, mouse, 1:1000, RRID: AB____627673) was added to 500 µl of protein lysis buffer containing 600 µg of protein lysates, and the reaction was maintained overnight at 4 °C on a rotator. The biological nanosurface technology was used to capture the immunocomplex. That is, the reaction was incubated with 30 µl of protein A/G magnetic beads (B23202, RRID: AB_1651895, Bimake, Houston, TX) on an orbital shaker for 2 hr at 4 °C. Then, protein-bead complex was separated from nonbinding proteins in the supernatant with a magnetic separator and then kept in 4 °C following rinsing. The supernatant was resuspended and incubated with 10 µl magnetic beads at room temperature for 30 min to capture the remaining immunocomplex. All the beads were pooled together and the supernatant was removed. The beads were resuspended in 2 × sample buffer (25 µl, P0015B, Beyotime, Shanghai, China), heated for 10 min at 100 °C to dissociate and denature the immunocomplex, and then spun down to collect supernatant for running Western blots as described earlier.

### Immunohistochemistry

The methods were basically the same as previously described (Wang and Hatton, 2009). For *in vivo* experiment, brains were decapitated and dissected in ice-cold ACSF and then fixed in 4% paraformaldehyde for 72 hr. The hypothalamic sections were cut into 70 µm-thick slices with a microtome. For *in vitro* experiment, treated brain slices were fixed with 4% paraformaldehyde for 4 hr at the room temperature. The fresh hypothalamic sections and treated brain slices were first permeabilized with 0.3% Triton X-100 in phosphate-buffered saline (PBS) for 1 hr and then treated with 0.5% bovine serum albumin in PBS for 1 hr to block nonspecific binding sites. Then, brain slices were incubated at 4 °C overnight with primary antibodies against pERK1/2 (OM125780, rabbit, 1:400), vasopressin-neurophysin (MabN 845, mouse, 1:2500, Merck Millipore, Shanghai, China, RRID: AB__2819363), GFAP (ab134436, chicken, 1:1000, UK, RRID: AB__2818977), or AQP4 (CST59678, rabbit, 1:1000, Cell Signaling Technology, Danvers, MA). After rinsing, brain slices were incubated with fluorescent donkey antibodies against mouse IgG H&L (ab150109, 1;1000, RRID: AB____2571721), rabbit IgG H&L (ab150062, 1;1000, RRID: AB____2801638), and chicken IgY H&L (ab150175, 1;1000, RRID: AB____2732800), respectively, for 2 hr at room temperature. Finally, Hoechst (CAS#28718-90-3, Sigma-Aldrich, Shanghai, China; 0.5 mg/ml, 30 min) was used to label the nuclei. Sections were examined with a fluorescence microscope (Eclipse FN1, Nikon, Tokyo, Japan) through a charge-coupled device camera (DS Ri2, Nikon, Tokyo, Japan) and then a confocal microscope (Eclipse Ti, Nikon, Tokyo, Japan). The sections/slices from different groups were taken from the same site/section of the SON to avoid bias due to site-associated difference in the distribution of neurons and astrocytes. To eliminate nonspecific staining, no-primary and no-secondary antibody reactions were applied to rule out false positivity.

### Data Collection and Analysis

In analyzing MCAO effects, we focused on events at MCAO 8 hr. This is because permanent MCAO for 4 to 12 hr is a critical window from the reduction to degeneration of the microvascular basement membrane-astrocyte contact in rat ischemic basal ganglia (Kwon et al., 2009). In counting the number of vasopressin neurons in the SON, only those that had a somatic diameter larger than 20 µm were included. To measure the length of GFAP filaments, we counted GFAP filaments that were 50 µm or longer and 2 µm or wider. To assay GFAP expression in single optical section, whole frame of the image was compared on the same background level of fluorescence intensity. This single optical section-based analysis is sufficient for reflecting GFAP plasticity as validated in three-dimensional reconstructions and fluorescent microscopy (Wang and Hatton, 2009).

Statistical analyses were performed with SigmaStat program (SPSS, Chicago, IL). All data were expressed as mean ± SEM or percentage of controls. Student’s *t* test was used for the analysis of differences between two groups. Analysis of variance (ANOVA) were used for analyzing three or more groups, and followed by Bonferroni test and Dunnett’s T3 test for normally distributed variables and for not normally distributed variables, respectively. *p* < .05 was considered statistically significant.

## Results

In general, sham rats did not show severe neurological index, positive TTC staining of the cerebral cortex or significant change in brain water contents. By contrast, MCAO rats showed obvious damages of the cerebral cortex accompanying with severe neurological index. Microinjection of TGN-020 in the SON partially reduced the brain damage (Figure 1A).

### Effects of MCAO on Brain Water Contents

Increasing brain water content is a key parameter of brain edema in ischemic stroke. However, MCAO for 8 hr did not significantly change cerebral water contents as a whole brain because different brain regions had different water contents. Thus, we dissected the cerebral cortex (excluding frontal and occipital lobes) into four zones (Figure 1, Inset). That is, left lateral/temporal (Zone A, non-MCAO side), left medial/parietal (Zone B, non-MCAO side), right medial/parietal (Zone C, MCAO side), and right lateral/temporal (Zone D, MCAO side, infarct core) zones. In sham rats, there was no significant difference in brain water contents between different zones in rats with ACSF or TGN-020 microinjection (Figure 1B). That is, the water contents in Zones A, B, C, and D in ACSF + sham were 80.7 ± 0.25%, 80.8 ± 0.16%, 80.7 ± 0.24%, and 81.1 ± 0.09%, respectively (*n* = 6; *p* > .05 by ANOVA; Figure 1Ba). Similarly, in TGN-020 + sham group, the water contents in Zones A, B, C, and D were 80.9 ± 0.25%, 79.6 ± 0.26%, 79.8 ± 0.25%, and 80.5 ± 0.22%, respectively (*n* = 6, *p* > .05 by ANOVA; Figure 1Bc). By contrast, MCAO selectively increased brain water contents in Zone D and microinjection of TGN-020 did not significantly change MCAO-evoked increase in water content of Zone D (Figure 1Be). The water contents at Zones A, B, C, and D of ACSF + MCAO group were 80.9 ± 0.2%, 80.0 ± 0.23%, 81.1 ± 0.23%, and 83.6 ± 0.3%, respectively (*n* = 6, *p* < .005 between Zone D and any of the other three zones by Dunnett’s T3 test; Figure 1Bb). TGN-020 + MCAO did not significantly change the trend of MCAO-evoked increase in brain water content in Zone D (Figure 1Be). That is, water contents at Zones A, B, C, and D were 81.0 ± 0.1%, 79.8 ± 0.1%, 80.5 ± 0.3%, and 82.9 ± 0.4%, respectively (*n* = 6, *p* < .005～<0.05 between Zone D and any of the other three zones by Dunnett’s T3 test; Figure 1 Bd).

### Effects of MCAO on pERK1/2 Expression in the SON

**Figure 2.**
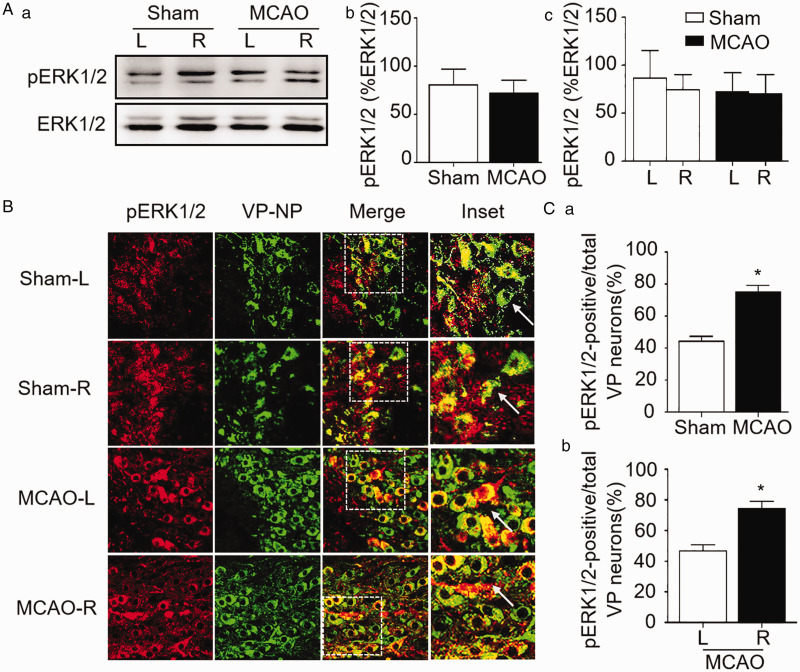
Effects of MCAO on Phosphorylated Extracellular Signal-Regulated Protein Kinase 1/2 (pERK1/2) Expression in the SON. A: Representative Western blotting bands (a) and bar graphs showing the effects of MCAO on pERK1/2 expression in whole SON (b) and at different sides in sham and MCAO rats (c). B: Effects of MCAO on pERK1/2 distribution in the SON in representative fluorescent images of pERK1/2 (in red), vasopressin-neurophysin (VP-NP in green), merges of pERK1/2 + VP-NP, and the enlarged inset from the white squares. C: Bar graphs summarizing the effects of MCAO on the percentage of pERK1/2-positive VP neurons over total VP neurons in the SON of the sham versus MCAO (a) and MCAO side (MCAO-R) versus non-MCAO side (MCAO-L) (b). **p < .01 comparing between the two groups by *t* test (Ca); ##*p* < .01 compared in MCAO group by paired *t* test (Cb). Annotations refer to Figure 1.

Vasopressin in the blood and brain is released from vasopressin neurons upon their activation (Jia et al., 2016). After determining that MCAO time-dependently increased plasma vasopressin levels (i.e., 119.3 ± 21.2% at 2 hr; 184.2 ± 23.8% at 8 hr, and 225.9 ± 18.5% at 24 hr relative to sham 2 hr, *n* = 4–8, *p* < .05 by ANOVA) in our preliminary study (Cui et al., 2019), we further analyzed pERK1/2 expressions in the SON to establish a causal association between the activity of vasopressin neurons and plasma vasopressin levels in MCAO rats. As shown in Figure 2A, pERK1/2 expression in Western blots did not change significantly in MCAO group compared with the sham group (80.3 ± 16.0%, *n* = 12 in sham vs.71.4 ± 13.4%, *n* = 12 in MCAO, *p* = .671 by *t* test). Moreover, there was no significant difference between bilateral SON of the sham rats (86.5 ± 29.1%, *n* = 6 in the left side vs.74.2 ± 16.1%, *n* = 5 in the right side, *p* = .441 by paired *t* test) and of MCAO rats (72.4 ± 19.8% in non-MCAO side vs. 70.4 ± 20.0% in MCAO side, *n* = 5, *p* = .824 by paired *t* test).

To determine whether different types of SON neurons expressed pERK1/2 differently, we further analyzed pERK1/2 expression in immunohistochemistry. The result showed that the number of pERK1/2-positive vasopressin neurons in the SON of MCAO rats was significantly higher than that in the sham-operated rats (74.9 ± 4.3%, *n* = 3 in MCAO vs. 44.2 ± 3.0%, *n* = 3 in sham, *p* < .01 by *t* test; Figure 2Ca). Moreover, the number of pERK1/2-positive vasopressin neurons was significantly higher in the SON of MCAO side than the non-MCAO side (46.9 ± 3.9%, *n* = 3 in non-MCAO side vs. 74.4 ± 4.8%, *n* = 3 in MCAO side, *p* < .01 by paired *t* test; Figure 2Cb).

### Effect of Microinjection of TGN-020 in the SON on pERK1/2 Expression in Zone D of the Cortex in MCAO Rats

**Figure 3.**
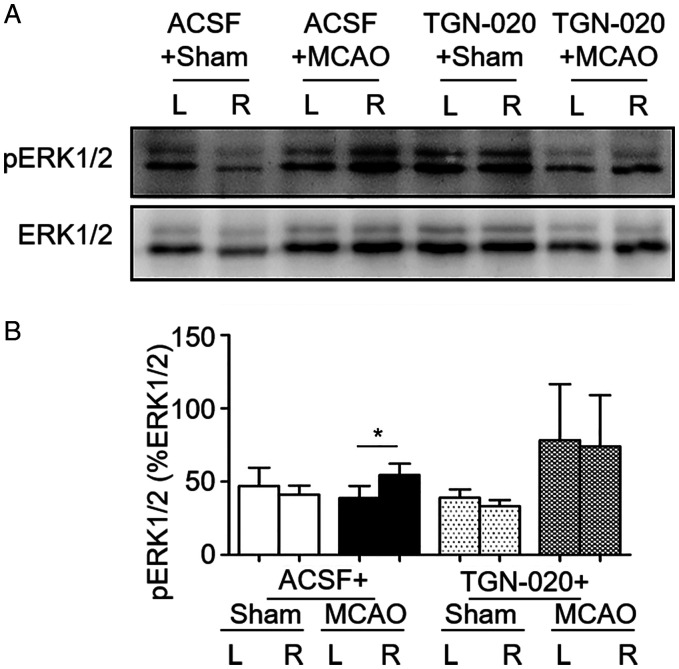
Effects of MCAO on Phosphorylated Extracellular Signal-Regulated Protein Kinase 1/2 (pERK1/2) Expressions After Blocking Aquaporin4 With N-(1,3,4-Thiadiazolyl) Nicotinamide (TGN-020) in the Supraoptic Nucleus. A: Representative Western blotting bands. B: Bar graph summarizing the expression of pERK1/2 in the cortex at different sides (b). *p < .05 compared between bilateral side by paired *t* test. Annotations refer to Figure 1 and 2.

Ischemic brain injury is associated with increased pERK1/2 signaling (Z. Q. Wang et al., 2004). If ischemic brain injury results from the activation of vasopressin neurons through maladapted astrocytic plasticity, inhibition of their activity by blocking abnormal astrocytic plasticity in the SON should reduce pERK1/2 expression in the cortex. To test this hypothesis, we examined pERK1/2 expressions in Zone D in MCAO rats without and with TGN-020 microinjection in the SON in Western blots. In sham group, there was no significant difference in pERK1/2 levels between bilateral sides of the cerebral cortex (46.8 ± 12.6%, *n* = 5 in Zone A vs. 41.0 ± 6.2%, *n* = 5 in Zone D, *p* = .607 by paired *t* test; Figure 3Ab). In MCAO rats, MCAO side expressed more pERK1/2 than the non-MCAO side (54.5 ± 7.6%, *n* = 4 in Zone D vs. 38.7 ± 8.3%, *n* = 4 in Zone A, *p* =.018 by paired *t* test; Figure 3 Ab). In sham rats, microinjection of TGN-020 in the SON remained no significant effect on pERK1/2 expression between bilateral cortex (38.9 ± 5.7%, *n* = 5 in Zone A vs. 33.1 ± 4.3%, *n* = 5 in Zone D, *p* = .292 by paired *t* test; Figure 3B). However, MCAO-evoked bilateral difference in pERK1/2 expressions in the cortex became insignificant after microinjection of TGN-020 in the SON (74.1 ± 34.8%, *n* = 4 in Zone D vs. 78.2 ± 38.2%, *n* = 4 in Zone A, *p* =.95 by paired *t* test; Figure 3B).

**Figure 4.**
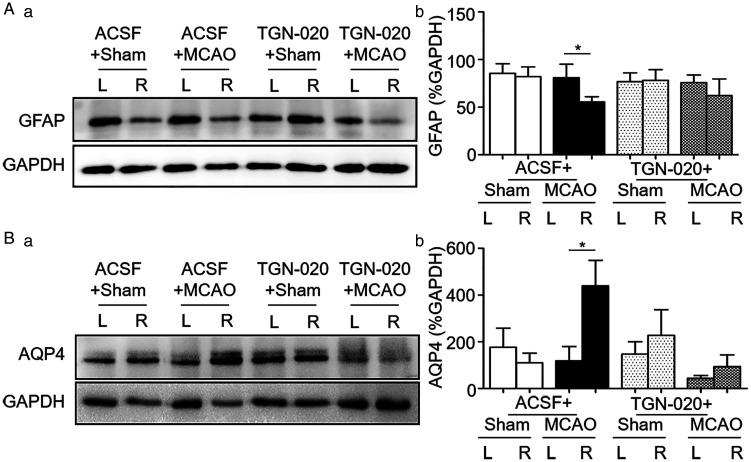
Effects of MCAO on Glial Fibrillary Acidic Protein (GFAP) and Aquaporin 4 (AQP4) Expressions After Blocking AQP4 With N-(1,3,4-Thiadiazolyl) Nicotinamide (TGN-020) in the Cortex. A and B: Representative Western blotting bands (a) and bar graphs (b) summarizing the expression of GFAP (A) and AQP4 (B) in the cortex of sham and MCAO rats. * *p* < .05 compared between bilateral sides by paired *t* test. Annotations refer to Figure 1.

### Effect of Microinjection of TGN-020 in the SON on GFAP Expression in Zone D of MCAO Rats

GFAP is an indicator of astrocytic plasticity; retraction of astrocytic processes or GFAP filaments from the surrounding of neurons is pivotal for the development of ischemic brain injury (Wang and Parpura, 2016, 2018). If blocking abnormal astrocytic plasticity in the SON can reduce vasopressin secretion, it should also block GFAP reduction in Zone D of the cortex. To test this hypothesis, we compared bilateral GFAP expressions in the cortex of MCAO rats without and with microinjection of TGN-020 in the SON. As shown in Figure 4Aa, there was no significant difference between bilateral cortex in sham rats (85.4 ± 10.2%, *n* = 6 in Zone A vs. 82.0 ± 10.3%, *n* = 6 in Zone D, *p* = .694 by paired *t* test; Figure 4Ab). In MCAO rats, GFAP expression in the MCAO side was significantly lower than the non-MCAO side (55.6 ± 13.2%, *n* = 6 in Zone D vs. 80.9 ± 35.0%, *n* = 6 in Zone A, *p* = .047 by paired *t* test; Figure 4Ab).

### Effect of Microinjection of TGN-020 in the SON on AQP4 Expressions in Zone D of MCAO Rats

AQP4 is a major target of vasopressin in ischemic stroke-evoked brain edema injury (Wang and Parpura, 2016, 2018). Thus, we further examined AQP4 expression in Zone D without and with microinjection of TGN-020 into bilateral SON before establishing MCAO model (Figure 4Ba). The results showed that there was no significant difference in AQP4 expression between the two sides of the cortex in the sham group (177.2 ± 81.6%, *n* = 5 in Zone A vs. 109.6 ± 41.7%, *n* = 5 in Zone D, *p* = .310 by paired *t* test; Figure 4Bb). In MCAO rats, AQP4 expression was significantly higher in the MCAO side than that of non-MCAO side (439.9 ± 108.5%, *n* = 4 in Zone D vs. 118.5 ± 60.8%, *n* = 4 in Zone A, *p* = .02 by paired *t* test; Figure 4Bb).

After blocking AQP4 with intra-SON microinjection of TGN-020, bilateral difference in AQP4 expression in sham rats remained insignificant (147.7 ± 53.1%, *n* = 5 in Zone A vs. 227.8 ± 109.4%, *n* = 5 in Zone D, *p* = .232 by paired *t* test; Figure 4Bb). However, application of TGN-020 in the SON made the difference in bilateral AQP4 expressions in MCAO rats insignificant (94.1 ± 50.1%, *n* = 4 in Zone D vs. 44.9 ± 10.8%, *n* = 4 in Zone A, *p* = .351 by paired *t* test; Figure 4Bb).

### Effects of OGD on GFAP and AQP4 Expressions in the SON in the Presence of TGN-020 or Phloretin

**Figure 5.**
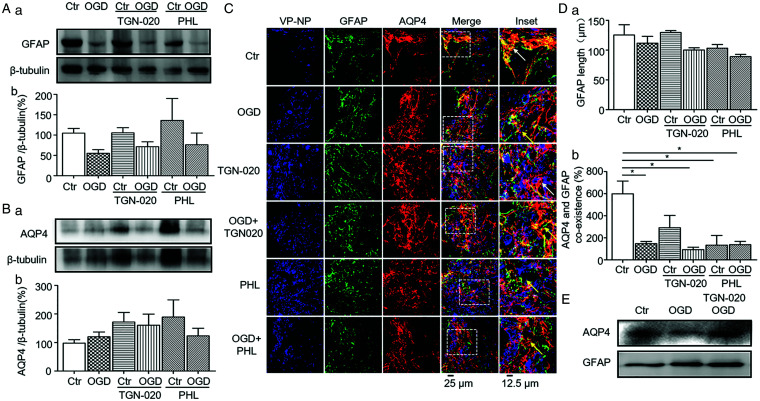
Effects of N-(1,3,4-Thiadiazolyl) Nicotinamide (TGN-020) and Phloretin on Glial Fibrillary Acidic Protein (GFAP) and Aquaporin 4 (AQP4) Expressions in the Supraoptic Sucleus Under Oxygen and Glucose Deprivation (OGD). A and B: Representative Western blotting bands (a) and bar graphs (b) summarizing the expression of GFAP (A) and AQP4 (B) in the SON. C: Representative confocal microscopic images of the immunohistochemistry of the SON showing vasopressin-neurophysin (VP-NP, in blue), GFAP (in green), AQP4 ( in red), merges and insets. D: Bar graphs summarizing the effects of TGN-020 and phloretin on GFAP filament length (a) and the percentage of colocalized GFAP filaments and AQP4 puncta surrounding VP neurons (b). *p < .05 between the two groups by Bonferroni test. E: Representative WB bands showing the effects of TGN-020 on OGD-associated molecular association between GFAP and AQP4 in the SON in hypothalamic brain slices. Annotations refer to Figures 1 and 2.

MCAO reduces blood irrigation of the SON in rats (Curras-Collazo et al., 2002). Thus, the SON could be influenced by ischemia directly. In our preliminary study, blocking astrocytic retraction with TGN-020 also blocked MCAO-evoked c-Fos expression in vasopressin neurons (Cui et al., 2019). Thus, astrocytes could be a critical modulator of vasopressin neuronal activity. To test this possibility, we further examined the effects of blocking AQP4 by TGN-020 and astrocytic volume-regulated anion channels by phloretin on the expression of GFAP and AQP4 in the SON under OGD. In Western blots (Figure 5A), OGD significantly reduced the expression of GFAP (104.8 ± 11.4% in control, *n* = 5 vs. 55.2 ± 9.0% in OGD, *n* = 5, *p* = .101 to control by Dunnett’s T3 test and *p* = .012 by paired *t* test; Figure 5Ab); however, there was no significant effect of OGD on the expression of GFAP in the presence of TGN-020 (105.5 ± 12.8% in TGN-020, *n* = 6, *p* = 1.0 to control by Dunnett’s T3 test; 71.7 ± 11.8% in OGD + TGN-020, *n* = 6, *p* = .547 to control by Dunnett’s T3 test; *p* = .175 between TGN-020 and OGD + TGN-020 by paired *t* test) or phloretin (135.7 ± 54.2% in phloretin, *n* = 6, *p* = 1.0 to control by Dunnett’s T3 test; 76.3 ± 28.7% in OGD + phloretin, *n* = 6, *p* = .991 to control by Dunnett’s T3 test; *p* = .399 between phloretin and OGD + phloretin by paired *t* test; Figure 5Ab).

By contrast, the expression of AQP4 did not change significantly in response to OGD (97.9 ± 12.4% in control, *n* = 6 vs. 55.2 ± 9.0% in OGD, *n* = 6, *p* = .979 by Dunnett’s T3 test and *p* = .129 by paired *t* test; Figure 5Bb), TGN-020, OGD + TGN-020 (171.8 ± 33.5% in TGN-020 group, *n* = 6, *p* = .532 to control by Dunnett’s T3 test; 160.7 ± 38.7% in OGD + TGN-020 group, *n* = 6, *p* = .810 to control by Dunnett’s T3 test; *p* = .682 between TGN-020 and OGD + TGN-020 by paired *t* test; Figure 5Bb), phloretin and OGD + phloretin (189.4 ± 59.5% in phloretin group, *n* = 6, *p* = .825 to control by Dunnett’s T3 test; 123.2 ± 26.6% in OGD + phloretin group, *n* = 6, *p* =.995 to control by Dunnett’s T3 test; *p* = .348 between phloretin and OGD + phloretin by paired *t* test; Figure 5Bb).

Then, we analyzed the distribution of GFAP and AQP4 surrounding vasopressin neurons in the SON using immunohistochemistry (Figure 5C). The length of GFAP in the SON was not influenced by OGD, TGN-020, or phloretin (*n* = 3). In the presence of TGN-020 or phloretin, OGD did not significantly influence the length of GFAP filaments as well (125.5 ± 17.2 µm in control group, *n* = 3 vs. 111.7 ± 11.3 µm in OGD group, *n* = 3, *p* = 1.0 by Bonferroni test; 129.7 ± 3.1 µm in TGN-020 group, *n* = 3, *p* = 1.0 to control by Bonferroni test; 100.2 ± 3.8 µm in OGD + TGN-020 group, *n* = 3, *p* = .961 to control by Bonferroni test; 103.1 ± 6.3 µm in phloretin group, *n* = 4, *p* = 1.0 to control by Bonferroni test; 89.1 ± 3.7 µm in OGD + phloretin group, *n* = 4, *p* = .121 to control by Bonferroni test; Figure 5 Da).

Further analyzing the colocalization of GFAP with AQP4 in the SON (Figure 5Db) revealed that OGD as well as phloretin significantly reduced their colocalization (600 ± 114% in control, *n* = 5 vs. 146.7 ± 20.2% in OGD, *n* = 5, *p* = .009 by Bonferroni test; 133.3 ± 88.2% in phloretin, *n* = 4, *p* = .026 compared with control by Bonferroni test). This is in agreement with the opposite trend of GFAP and AQP4 expression at protein levels. Notably, OGD-evoked reduction in the colocalization became insignificant (*p* > .05 by *t* test) in the presence of TGN-020 (291.7 ± 111.4% in TGN-020, *n* = 6, *p* = .144 to control by Bonferroni test; 93.8 ± 21.3% in OGD + TGN-020, *n* = 4, *p* = .006 to control by Bonferroni test) or phloretin (133.3 ± 88.2% in phloretin, *n* = 3, *p* = .026 to control by Bonferroni test; 138.3 ± 29.1% in OGD + phloretin, *n* = 5, *p* = .008 to control by Bonferroni; Figure 5Db).

Finally, to establish functional association between GFAP and AQP4, we analyzed the molecular association between GFAP and AQP4 under the OGD condition. Co-immunoprecipitation confirmed the existence of molecular association between GFAP and AQP4 (Figure 5E). Moreover, OGD significantly reduced the molecular association of GFAP with AQP4, which was attenuated by pretreatment with TGN-020. This finding is in agreement with the reduced colocalization between GFAP and AQP4 as well as the protective effect of TGN-020 in the SON on MCAO-evoked brain injuries.

## Discussion

**Figure 6.**
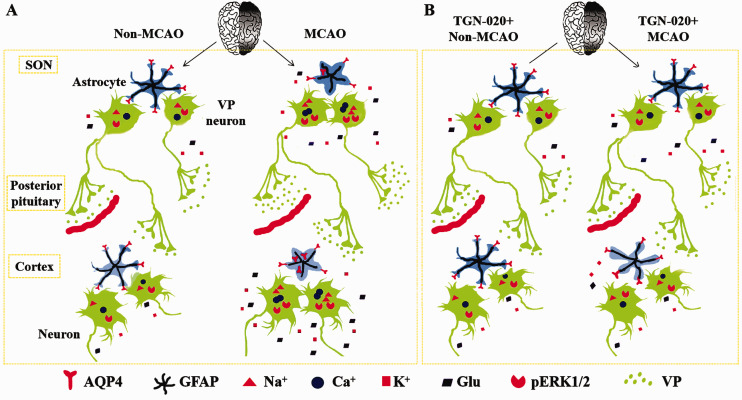
Diagrammatic Drawings of MCAO-Evoked Malfunctions of Supraoptic Astrocytes, VP Hyperactivation, Cerebral Damages, and the Preventive Effect of Blocking AQP4 in the SON. A: MCAO. B: Inhibition of AQP4 before MCAO. AQP4 = aquaporin 4; GFAP= glial fibrillary acidic protein; MCAO = middle cerebral artery occlusion; pERK1/2 = phosphorylated extracellular signal-regulated protein kinase1/2; SON = supraoptic nucleus; TGN-020 = N-(1,3,4-Thiadiazolyl)nicotinamide; VP = vasopressin.

MCAO can increase vasopressin neuronal activity in the SON, specifically at the MCAO side. This effect is associated with discoordinated reduction of GFAP with AQP4 and the subsequent retraction of astrocytic processes evoked by OGD in the SON. Blocking the retraction of astrocytic processes by microinjection of TGN-020 into the SON can alleviate MCAO-evoked increases in pERK1/2 and AQP4 and the decrease in GFAP expression in the cerebral cortex of MCAO side (Figure 6). These findings indicate that ischemic stroke-associated vasopressin hypersecretion is correlated with the activation of vasopressin neurons and brain damage and that blocking abnormal astrocytic plasticity in the SON has the potential to alleviate brain injury by suppressing vasopressin hypersecretion.

### Ischemic Stroke and Vasopressin Secretion

Ischemic stroke can increase vasopressin secretion (Barreca et al., 2001; Chang et al., 2006). This is verified by the present finding that pERK1/2 expression in vasopressin neurons was increased in the SON. In the SON, MCAO significantly increased pERK1/2-positive vasopressin neurons although the total amount of pERK1/2 expression in Western blots did not change significantly. This contradictory phenomenon is likely due to the presence of multiple cell types in the SON, which makes the specific activation of vasopressin neurons averaged by the nonresponsive oxytocin neurons in Western blots in the SON of MCAO side. The activation of vasopressin neurons is in agreement with the retraction of astrocytic processes around them (Jia et al., 2016; Wang and Parpura, 2016).

The activation of vasopressin neurons can be achieved via multiple approaches as previously reviewed (Jia et al., 2016; Wang and Parpura, 2016). That is, at systemic level, vasopressin increase is attributable to the activation of sympathetic nervous system and the renin-angiotensin-aldosterone system. In the brain, spreading depolarization wave from the infarct core can activate vasopressin neurons due to increases in extracellular glutamate and K^+^ levels. In the SON, OGD-evoked malfunction of astrocytes may directly increase vasopressin neuronal activity. This can be achieved by evoking regulatory volume decrease of astrocytes. This possibility is supported by the finding that MCAO decreased GFAP expression in the SON, which specifically occurred at the MCAO side.

Also as previously reviewed (Jia et al., 2016; Wang and Parpura, 2016), OGD in ischemic stroke can cause the failure of sodium pump that relies on adenosine triphosphate production through oxidative phosphorylation and thus results in cellular swelling due to sodium retention. Cell swelling further activates volume-regulated anion channels, which allows astrocytes to release Cl^−^ and organic anions while reduces absorption of extracellular glutamate and K^+^. As a result, vasopressin neuronal activity and vasopressin release are increased.

### Vasopressin and Brain Injury

In general, OGD, reperfusion injury, and vasopressin hypersecretion are considered as the main causes of brain injury (O'Donnell et al., 2013), particularly vasopressin hypersecretion (Jia et al., 2016; Wang and Parpura, 2016). Vasopressin can increase the activity of Na^+^, K^+^, 2Cl^−^, and water co-transporter 1 and endothelial hydrogen-sodium exchanger, thereby increasing ion transport across the blood–brain barrier and extracellular edema (Hockel et al., 2012). Activation of the V1a receptor also increases brain endothelin 1 and thus increases brain susceptibility to hypoxia and vasomotor dysfunction (Faraco et al., 2014). As a result, integrity of the blood–brain barrier is disrupted.

This study revealed that MCAO increases the expressions of pERK1/2 and AQP4 protein levels in the cerebral cortex in the MCAO side. The increase in pERK1/2 in Zone D can reflect the activation of pro-inflammatory cytokine interleukin-1β signaling, which induces an inflammatory response and exacerbates ischemic brain injury (Z. Q. Wang et al., 2004). Consistently, blocking ERK1/2 activation using U0126 can block focal ischemic stroke-evoked brain injury (Namura et al., 2001; Z. Q. Wang et al., 2004) and reduced pERK1/2 expression is associated with neuroprotective effect of isoflurane preconditioning against MCAO in the rat (Zhao et al., 2019). By contrast, the increased AQP4 expression is also an index of increased brain water contents (Wang and Parpura, 2016) and is in agreement with the increase in water contents in Zone D of MCAO rats. It remains to clarify whether increased pERK1/2 and AQP4 occur in the same cells or different cell population in Zone D.

Obviously, these effects are mediated by vasopressin hypersecretion from vasopressin neurons. The blocking effects of TGN-020 in the SON on cerebral damages in the MCAO side indicate the involvement in neural innervation of vasopressin neurons rather than nonspecific diffusion as further discussed. Thus, vasopressin hypersecretion plays a key role in the development of ischemic pathogenesis and blocking vasopressin hypersecretion has the potential to reverse ischemic brain injury.

### Association of Abnormal Astrocytic Plasticity Between the Cerebral Cortex and the SON in MCAO

MCAO increased AQP4 and the decreased GFAP expressions in Zone D of the MCAO side. The increase in AQP4 expression is in agreement with the increased cerebral water contents in Zone D; the decrease in GFAP expression corresponds to the finding that astrocytes in the infarct core are disrupted in ischemic stroke. By contrast, MCAO-evoked reduction in GFAP expressions suggests the occurrence of regulatory volume decrease and its toxic influence on neural tissues in the infarct zone as previously reviewed (Jia et al., 2016; Wang and Parpura, 2016).

Under physiological condition, AQP4 activity, together with GFAP plasticity, determines the transfer of water between neural tissues and the blood (Pekny and Pekna, 2004; Wang and Parpura, 2016). The reduction of GFAP, an indicator of removal of astrocytic endfeet from the surrounding of the blood vessels, indicates disruption of the blood–brain barrier. This event can coexist with depolarization of AQP4 from astrocytic endfeet and thus, water fails to be transferred into the blood normally. The increased AQP4 during the retraction of GFAP filaments/astrocytic processes likely promotes water efflux from astrocytes to accumulate in the extracellular space. At the neural microenvironment, removal of astrocytic processes disables their ability to buffer extracellular K^+^ and glutamate through AQP4 in the infarction area, thereby causing excitotoxicity of neurons in the ischemic regions. Resultantly, brain edema worsens over time. This finding is consistent with previous reports that MCAO significantly increased cerebral expression of AQP4 (C. C. Wang et al., 2010) and brain edema was significantly reduced after inhibition of AQP4 expression in rats (S. Li et al., 2015).

Interestingly, TGN-020 in the SON also reduced MCAO-evoked increase in AQP4 and the decrease in GFAP expressions in Zone D. The effect of blocking AQP4 is also in agreement with the trend of reduction in cerebral water contents. It indicates alleviation of the negative influence of maladapted astrocytes, a finding consistent with the effect of TGN-020 in the SON on glycogen synthase kinase-3β expression in the cerebral cortex (Cui et al., 2019).

Notably, astrocytes in the SON and in the cerebral cortex have no direct structural connections. When TGN-020 is applied in the SON, a brain region far from the infarct core, it is unlikely for the drug to act on the cortex. Thus, the action of TGN-020 should be limited in the SON.

In the SON, astrocytes are structurally associated with both vasopressin and oxytocin neurons (Hou et al., 2016). Since oxytocin has protective effect against ischemia-evoked injury (Etehadi Moghadam et al., 2018; P. Wang et al., 2019), the protective effect of TGN-020 in the SON on the cortex should be attributable to reduced vasopressin but not oxytocin neuronal activity. This possibility is supported by the finding that MCAO increased pERK1/2 levels in the SON and plasma vasopressin (Cui et al., 2019). Since axon collaterals share the same vasopressin neuronal activity with axon terminals in the posterior pituitary (Hou et al., 2016), the increased plasma vasopressin level suggests increased vasopressin release from axon collaterals in the brain. In addition, that the abnormal cerebral astrocytic plasticity occurred in the same side of the SON with increased vasopressin neuronal activity also indicates that the pathological effect was mediated by neural approach but not via diffusion of vasopressin through circulation or cerebrospinal fluid.

The retraction of astrocytic processes can facilitate vasopressin release. As shown in the Results section, there was significant reduction of GFAP levels in OGD, suggesting retraction of astrocytic processes. This could result from the direct effect of OGD and its elicited regulatory volume decrease since phloretin also blocked OGD-evoked reduction of GFAP in the SON. This finding is consistent with the significant reduction in GFAP levels of the peri-infarct areas (Lively et al., 2011) and fragmentation of astrocytic processes in the hypothalamus (Abraham et al., 2003). The retraction can in turn facilitate vasopressin neuronal activity through increasing synaptic innervation, junctional coupling, cell apposition, and reducing reabsorption of extracellular neurochemical substances (Jia et al., 2016).

In conclusion, this study for the first time links vasopressin hypersecretion during ischemic stroke to the activation of vasopressin neurons in the SON due to OGD-evoked malfunction of astrocytic plasticity. Since the activation of vasopressin neuronal activity and vasopressin release into the brain and the blood have been implicated in many pathological processes including GFAP and AQP4-associated brain damages (Wang and Parpura, 2016, 2018; D. Li et al., 2020), clarification of how astrocytes in the SON regulate vasopressin neuronal activity will provide novel therapeutic target to prevent the occurrence the abnormal activity of astrocytes.
